# *Cyclocarya paliurus* tea leaves enhances pancreatic **β** cell preservation through inhibition of apoptosis

**DOI:** 10.1038/s41598-017-09641-z

**Published:** 2017-08-22

**Authors:** Hai-tao Xiao, Bo Wen, Zi-wan Ning, Li-xiang Zhai, Cheng-hui Liao, Cheng-yuan Lin, Huai-xue Mu, Zhao-xiang Bian

**Affiliations:** 1 School of Chinese Medicine, Hong Kong Baptist University, Kowloon Tong, Kowloon Hong Kong; 2 Shenzhen Research Institute and Continuing Education, Hong Kong Baptist University, Shenzhen, China

## Abstract

Leaves of *Cyclocarya paliurus* are a sweet tea traditionally used to treat obesity and diabetes in China. However, its protective mechanisms against hyperglycemia remains unclear. Here, we demonstrate that the extract of *C. paliurus* leaves significantly decreased body loss, food intake and blood glucose level, and increased blood insulin level, β-cell number and insulin-producing β cells in high-fat diet-low dose STZ-induced diabetic mice. *In vivo* and *in vitro* studies also showed the extract of *C. paliurus* leaves significantly inhibited pancreatic β cell apoptosis by suppressing the expression of caspase 8, caspase 9 and cleaved caspase-3, as well as Bax/Bcl-2 ratio, down-regulating p38, ERK and JNK phosphorylation, and up-regulating Akt phosphorylation. These effects were significantly enhanced by inhibitor p-38 or ERK or JNK, and counteracted by inhibitor of PI3K. In addition, the extract of *C. paliurus* leaves also significantly improved hepatic steatosis, nephropathy and cardiac hypertrophy of diabetic mice. Taken together, these results provide the insight into the effects of *C. paliurus* leaves on pancreatic β cell preservation in standing glucolipotoxicity. Therefore, *C. paliurus* tea leaves may be used as a new remedy for diabetes through enhancing pancreatic β cell preservation by inhibiting β cell apoptosis.

## Introduction

Type 2 diabetes mellitus (T2DM) is a chronic metabolic disorder characterized by a persistent increase in blood glucose above normal values (hyperglycemia). It is estimated that by 2035, 471 million people in the world will be affected by this illness, and it is becoming a global public health concern due to its high morbidity and mortality^1^. Although many aspects of T2DM remain unclear, it is generally recognized that peripheral insulin resistance and pancreatic β cell failure are two major pathological changes during the progression of T2DM^2^. Normal pancreatic β cells have a compensation mechanism to up-regulate insulin secretion and/or β cell mass, which can maintain normoglycemia when insulin resistance occurs. Once the functional pancreatic β cell mass decreases and fails to compensate for insulin resistance, T2DM would be developed^3, 4^. Accordingly, enhancing β cell preservation represents a novel and important therapeutic strategy to prevent or treat T2DM.

In Traditional Chinese Medicine (TCM) system, T2DM is known as “Xiao-Ke-Zheng” whose core pathogenesis is “*Yin* deficiency and dryness-heat”, and bitter and cold TCMs are usually recommended to remove heat and invigorate *Yin* according to the TCM theory, have demonstrated good antidiabetic effects *in vitro* and *in vivo*, as well as in clinical practice^5^. *Cyclocarya paliurus* (Batal) IIjinskajia, an endemic plant belonging to *Cyclocarya* genus of the Juglandaceae family, mainly distributed in the southern of China^6^. The leaves of *C. paliurus* (CP) has long been used as a bitter TCM with the property of clearing heat and toxin to treat obesity and diabetes, which has also been historically used as an herbal tea in the folk^7^. Based on its property, in 1999, antihyperglycemic herbal tea processed by leaves of *C. paliurus* has been approved by the United States Food and Drug Administration (FDA) as a dietary supplement product^7^. Nowadays, the products derived from the leaves of *C. paliurus* have become a very popular health product in China^6^. It has been reported that the leaves of *C. paliurus* are composed of various active components including flavonoids, phenolic acids, triterpenoids, carbohydrates and sterols^8^. Pharmacological investigations revealed that the leaves of *C. paliurus* could decrease blood glucose and increase insulin level of diabetic rats induced by streptozotocin (STZ)^9^. However, its antihyperglycemic effects and underlying mechanisms have not been well documented.

In this study, we used biomedical approaches to investigate the antidiabetic activity of the leaves of *C. paliurus*, as well as its underlying mechanisms in STZ-induced diabetic mice fed with a high-fat diet. Our data show that the leaves of *C. paliurus* exert a potent hypoglycemic effect to attenuate high-fat diet-low dose STZ-induced experimental type 2 diabetes in mice through inhibiting pancreatic β cell apoptosis.

## Results

### Phytochemical characteristics of CP extract

The extract of the leaves of *C. paliurus* was analyzed by UPLC obtaining the typical chromatogram (Supplementary Fig. 1). Compounds are identified in the chromatogram with peaks at 4.93 min to 1-caffeoylquinic acid, 7.00 min to 5-caffeoylquinic acid, 10 min to chlorogenic acid, 14.00 min to isoquercitrin, 16.00 min to kaempferol-3-glucoside, 17.00 min to kaempferol 3-rhamnoside, and 18.00 min to quercetin by comparison of the retention times (Rt) and ultraviolet (UV) absorption characteristics of external standards. As well, the contents of seven compounds in the extract of leaves of *C. paliurus* were measured by UPLC method and their contents were 1.4, 1.5, 4.0, 4.1, 1.6, 2.7 and 0.6 mg/g in the extract of leaves of *C. paliurus*, successively.

### CP extract alleviates STZ and high fat diet-induced diabetes in mice

**Figure 1:**
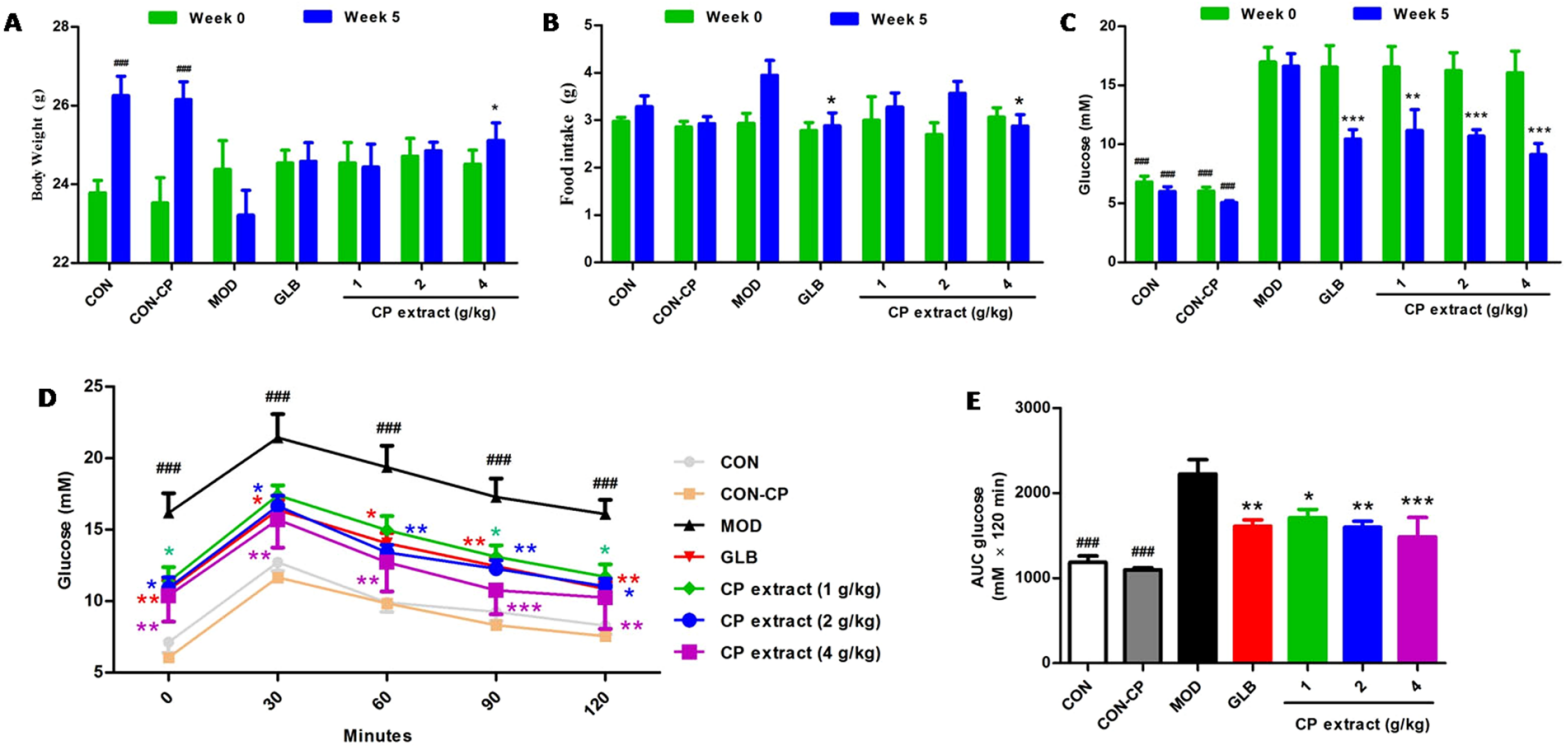
CP extract alleviates STZ and high fat-induced diabetes in mice. The type 2 diabetic mice were induced by feeding with high-fat diet for 4 weeks and then injecting intraperitoneally with 25 mg/kg STZ for 3 days consecutively. The diabetic mice with consecutive 7-day hyperglycemia (11 mmol/L or greater) were selected for the experiment and then CP extract or glibenclamide were administered to mice for consecutive 5 weeks. During the experiment, the body weight, food intake and blood glucose levels of mice were monitored weekly. The body weight on week 0 and 5 at checkpoint after the initiation of CP extract treatment respectively (**A**); The food intake on week 0 and 4 at checkpoint after the initiation of CP extract treatment respectively (**B**); The blood glucose levels on week 0 and 5 at checkpoint after the initiation of CP extract treatment respectively (**C**). The blood glucose levels (**D**) and the areas under curves (AUCs) constructed from blood glucose levels (**E**) of oral glucose tolerance test (OGTT) on week 4 at checkpoint after the initiation of CP extract treatment. The blood glucose levels in serum were determined by a glucometer (OMRON (China) Co., Ltd, Beijing, China). All data are presented as means ± SEM (n = 8). ^#^ *p* < 0.05, ^##^ *p* < 0.01 and ^###^ *p* < 0.001, diabetic model group compared with non-diabetic groups; ^*^ *p* < 0.05, ^**^ *p* < 0.01 and ^***^ *p* < 0.001, compared with diabetic model group. CON: non-diabetic control group; CON-CP: CP extract-treated non-diabetic control group; MOD: diabetic model group; GLB: glibenclamide-treated diabetic group.

To explore the protective and therapeutic roles of CP extract on high-fat diet and low dose of STZ-induced diabetes, the CP extract was orally administrated to diabetic mice for 5 weeks. As shown in Supplementary Fig. 2A, the body weight of diabetic mice was continual deline from the initiation of treatment, whereas this reduction was reversed by CP extract treatment in a dose-dependent manner. At the end of 5 week treatment, the body weight of diabetic mice in high dose of CP extract-treated group was much higher than that of mice in diabetic model group with a significant statistical difference (*P* < 0.05) (Fig. 1A **)**. In contrast, the food intake of diabetic mice was higher than that of non-diabetic mice, and also be rescued by CP extract- or glibenclamide treatment (Supplementary Fig. 2B). At checkpoint of 4 week after the initiation of treatment, the food intake of mice in CP extract- or glibenclamide treatment group was significantly lower than that of mice in diabetic group (*P* < 0.05) (Fig. 1B **)**. As reported elsewhere, the mice treated with high-fat diet with low dose of STZ resulted in a drastic elevation of blood glucose level, and this elevation was continually restricted by CP extract or glibenclamide treatment (Supplementary Fig. 1C). At the end of 5 week treatment, the level of blood glucose in CP extract or glibenclamide-treated diabetic group was much lower than that of diabetic model group with significant statistical differences (*P* < 0.001)(Fig. 1C **)**. To investigate the effects of CP extract on the development of diabetes, oral glucose tolerance test (OGTT) was conducted at checkpoint of 4 week after the initiation of CP extract treatment. As shown in Fig. 1D,E, after administration of glucose, the blood glucose levels of diabitic group significantly increased, as compared with non-diabetic groups, whereas the blood glucose levels of diabitic groups treated with CP extract- or glibenclamide decreased significantly. In addition, in the CP extract- or glibenclamide- treated diabitic groups, the areas under curves (AUCs) constructed from blood glucose levels were significantly decreased in comparison with that of diabitic group. These results suggested that CP extract has anti-hyperglycemic potential in high-fat diet-fed with low dose of STZ induced diabetic mice.

### CP extract prevents pancreatic β cell mass decrease in diabetic mice

**Figure 2:**
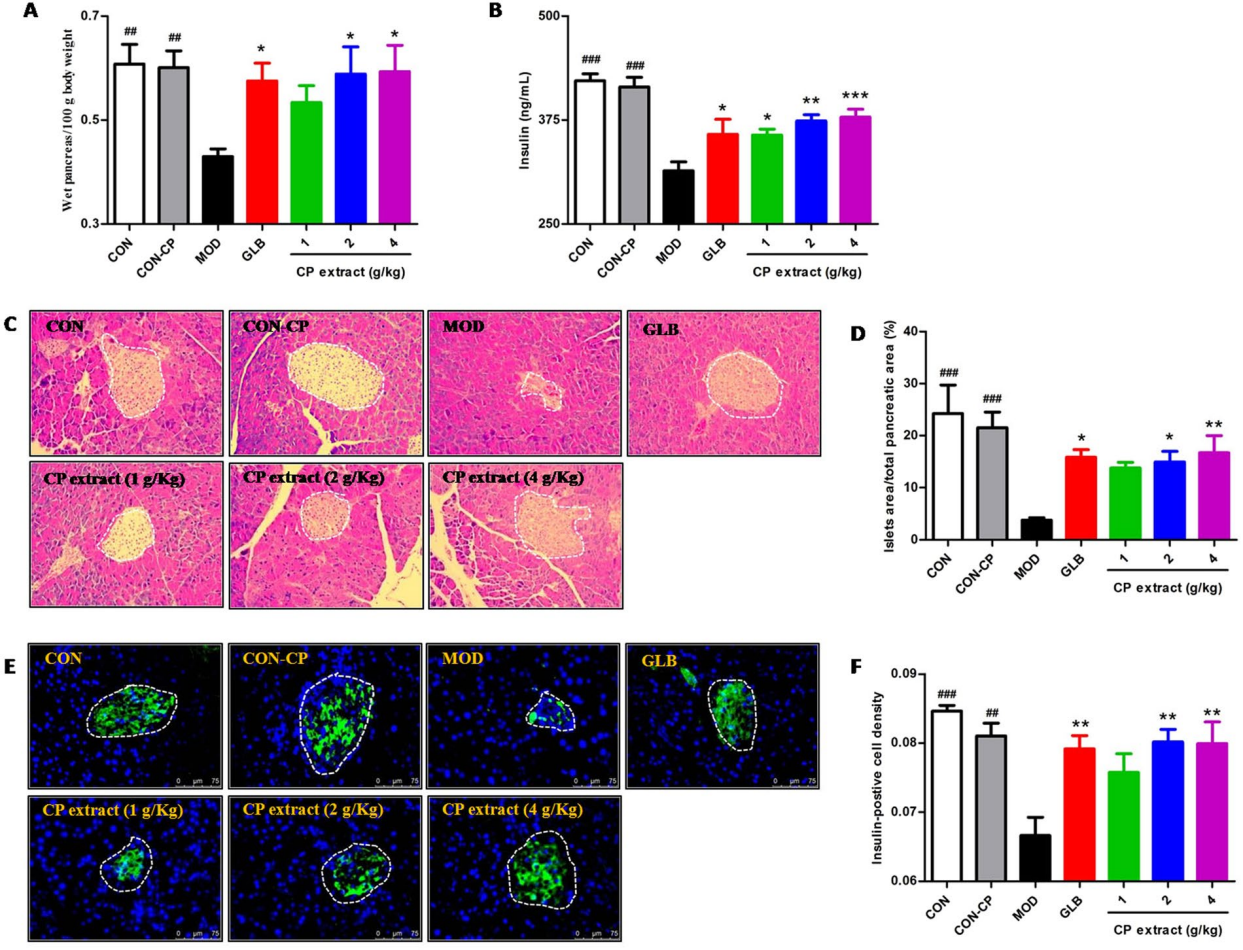
CP extract prevents pancreatic β cell mass decrease in diabetic mice. The type 2 diabetic mice were induced by feeding with high-fat diet for 4 weeks and then injecting intraperitoneally with 25 mg/kg STZ for 3 days consecutively. The diabetic mice with consecutive 7-day hyperglycemia (11 mmol/L or greater) were selected for the experiment and then CP extract or glibenclamide were administered to mice for consecutive 5 weeks. At the end of experiment, mice were sacrificed. The samples of blood and pancreas tissues were collected. (**A**) Relative weight of wet pancreas; (**C**) Histopathological examination of pancrease tissues (H & E staining) (magnification, 100×) and (**E**) Immunofluorescence (IF) analysis of insulin-positive cell using insulin marker (magnification, 600×). (**B**) Blood insulin levels; (**D**) Relative islets area in total pancreatic area; (**F**) Insulin-positive cell intensity. Blood insulin levels were assayed using commercially available kits (Wuhan Fine Biotech Co. Ltd, China). All data are presented as means ± SEM (n = 8). ^#^ *p* < 0.05, ^##^ *p* < 0.01 and ^###^ *p* < 0.001, diabetic model group compared with non-diabetic groups; ^*^ *p* < 0.05, ^**^ *p* < 0.01 and ^***^ *p* < 0.001, compared with diabetic model group. CON: non-diabetic control group; CON-CP: CP extract-treated non-diabetic control group; MOD: diabetic model group; GLB: glibenclamide-treated diabetic group.

To explore how the CP extract helped resist hyperglycemia, the pancreas relative weight, histopathological changes of pancreas, and serum insulin were detected. As shown in Fig. 2A, drastically decreased pancreas relative weight was observed in diabetic mice *vs*. control groups (*P* < 0.05). After treatment of CP extract, the pancreas relative weight was significantly reversed (*P* < 0.05). Histological examination of pancreatic sections revealed that the islet/pancreas area ratio in diabetic mice was significantly decreased in comparison with controls. In CP extract- or glibenclamide-treated groups, those decreases were largely alleviated (*P* < 0.05 and *P* < 0.01, respectively) (Fig. 2C,D). Subsequently, determination of insulin level in the serum of mice treated with STZ and high fat diet exhibited a drastic decrease, compared to control groups (*P* < 0.001), but this was significantly rescued after CP extract or glibenclamide treatment (*P* < 0.05 and *P* < 0.01, respectively) (Fig. 2B). Simultaneously, immunofluorescent-staining showed that the insulin-producing β-cells in the control group occupied a majority of the islet area, whereas staining was drastically diminished by treatment of STZ with high fat diet. This reduction was also prominently attenuated by the administration of CP extract or glibenclamide (*P* < 0.05 and *P* < 0.01, respectively) (Fig. 2E,F). These results indicate CP extract can preserve pancreatic islet β-cell mass.

### CP extract suppresses pancreatic β cell apoptosis in diabetic mice by modulating MAPK and Akt pathways

**Figure 3:**
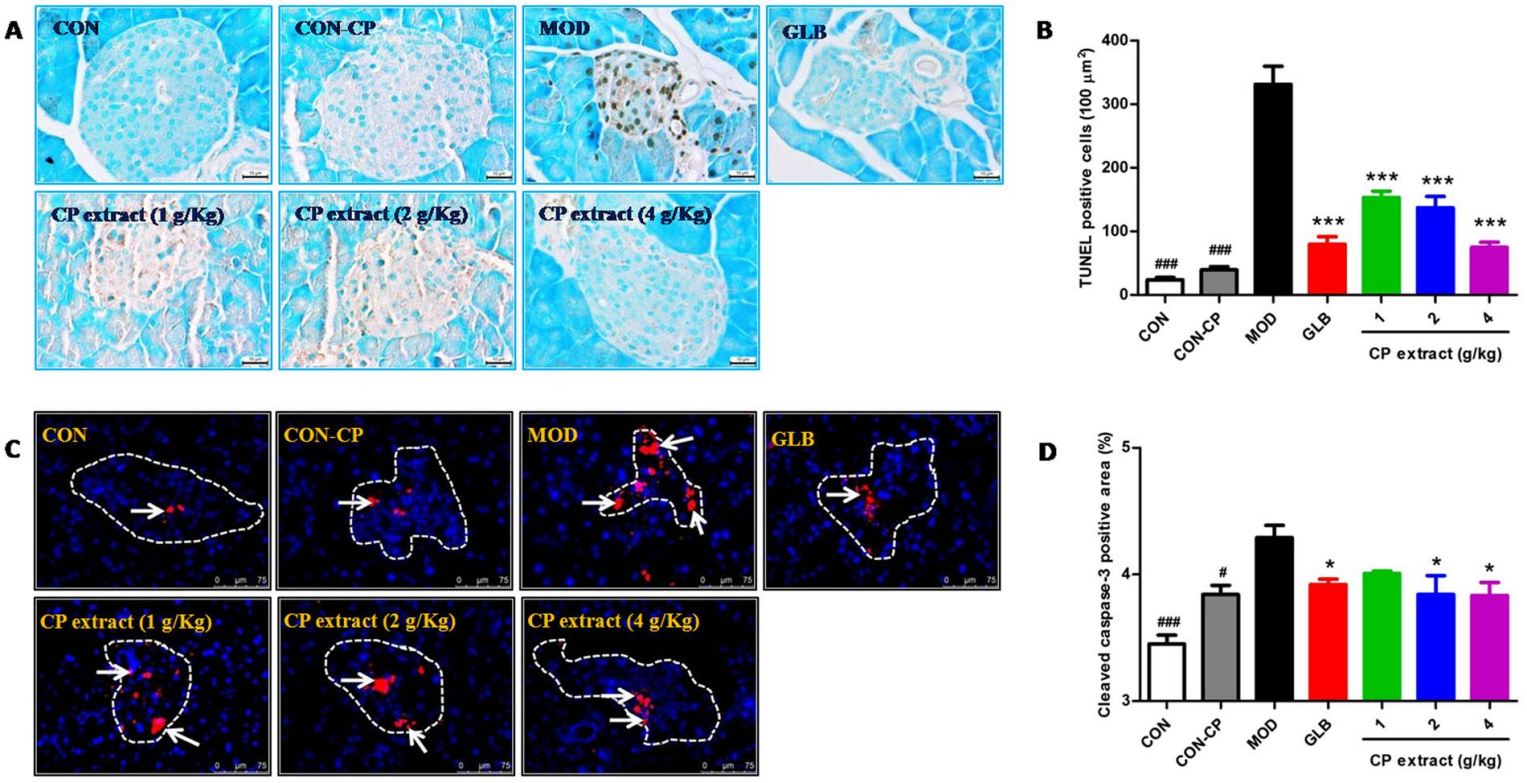
CP extract suppresses pancreatic β cell apoptosis in diabetic mice. The type 2 diabetic mice were induced by feeding with high-fat diet for 4 weeks and then injecting intraperitoneally with 25 mg/kg STZ for 3 days consecutively. The diabetic mice with consecutive 7-day hyperglycemia (11 mmol/L or greater) were selected for the experiment and then CP extract or glibenclamide were administered to mice for consecutive 5 weeks. At the end of experiment, mice were sacrificed. The samples of pancreas tissues were collected. (**A**) TUNEL staining of mice pancreas tissues (magnification, 600×) and (**C**) Immunofluorescence analysis of cleaved caspase-3 expression in mice pancreas tissues (magnification, 600×); (**B**) The number of TUNEL-positive cells; (**D**) Cleaved caspase-3-positive area. All data are presented as means ± SEM (n = 8). ^#^ *p* < 0.05, ^##^ *p* < 0.01 and ^###^ *p* < 0.001, diabetic model group compared with non-diabetic groups; ^*^ *p* < 0.05, ^**^ *p* < 0.01 and ^***^ *p* < 0.001, compared with diabetic model group. CON: non-diabetic control group; CON-CP: CP extract-treated non-diabetic control group; MOD: diabetic model group; GLB: glibenclamide-treated diabetic group.

**Figure 4:**
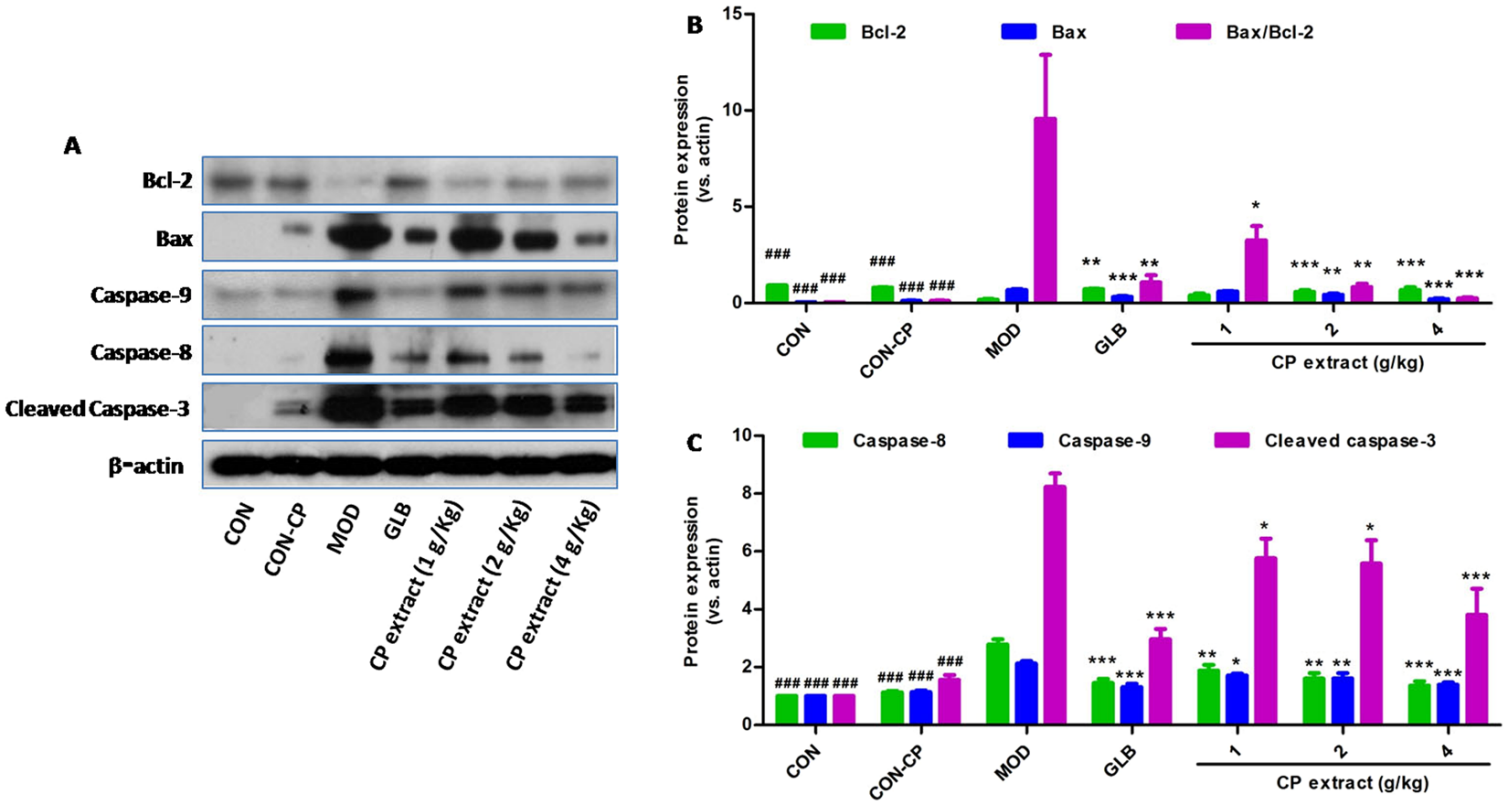
CP extract suppresses Bax/Bcl-2 ratio, caspase-8, Caspase-9 and cleaved caspase-3 in pancrease tissues of diabetic mice. The type 2 diabetic mice were induced by feeding with high-fat diet for 4 weeks and then injecting intraperitoneally with 25 mg/kg STZ for 3 days consecutively. The diabetic mice with consecutive 7-day hyperglycemia (11 mmol/L or greater) were selected for the experiment and then CP extract or glibenclamide were administered to mice for consecutive 5 weeks. At the end of experiment, mice were sacrificed. The samples of pancreas tissues were collected. Protein expressions of Bcl-2, Bax, caspase-8, Caspase-9 and cleaved caspase-3 were determined in pancreas tissues using Western blot. (**A**) Representative image of Western blot; (**B**) Protein expressions of Bcl-2, Bax and Bax/Bcl-2 ratio; (**C**) Protein expressions of caspase-8, Caspase-9 and cleaved caspase-3. All data are presented as means ± SEM (n = 8). ^#^ *p* < 0.05, ^##^ *p* < 0.01 and ^###^ *p* < 0.001, diabetic model group compared with non-diabetic groups; ^*^ *p* < 0.05, ^**^ *p* < 0.01 and ^***^ *p* < 0.001, compared with diabetic model group. CON: non-diabetic control group; CON-CP: CP extract-treated non-diabetic control group; MOD: diabetic model group; GLB: glibenclamide-treated diabetic group.

In the development of T2DM, pancreatic β cell apoptosis contributes to the loss of β cell mass^10^, we therefore detected β cell apoptosis in pancreatic tissues by TUNEL staining. As shown in Fig. 3A,B, the positive TUNEL staining signals in the pancreatic islets of diabetic mice were conspicuous, however, those signals were barely detected in the pancreatic islets of mice in the control group, indicating a major increase of apoptotic β cells in the pancreatic islets of diabetic mice. After treatment of CP extract or glibenclamide, positive TUNEL staining signals in the pancreatic islets of diabetic mice was significantly reduced (*P* < 0.001). Because caspase-3 activation has been implicated in pancreatic β cell apoptosis^11^, we also performed immunohistochemistry and Western blot analysis to detect cleaved active form of caspase-3. As compared with samples from the littermate control mice, the relative area of cleaved caspase-3-positive cells was significantly increased in pancreatic islet areas of the diabetic mice, whereas this increase was drastically diminished by treatment of CP extract or glibenclamide (*P* < 0.05) (Fig. 3C,D). As well, Western blot analysis also revealed that the expression of cleaved caspase-3 in pancreatic tissues of diabetic mice was robustly elevated, which was also significantly rescued after CP extract or glibenclamide treatment (*P* < 0.05 or *P* < 0.001). Consistent with elevated cleaved caspase-3, the expressions of apoptosis initiators caspase-8 and caspase-9, as well as Bax/Bcl-2 ratio in the pancreatic tissues of diabetic mice were significantly increased and those were significantly reversed in the presence of CP extract or glibenclamide (Fig. 4). Collectively, these results suggest a protective function of CP extract against apoptosis of pancreatic β cells.

**Figure 5:**
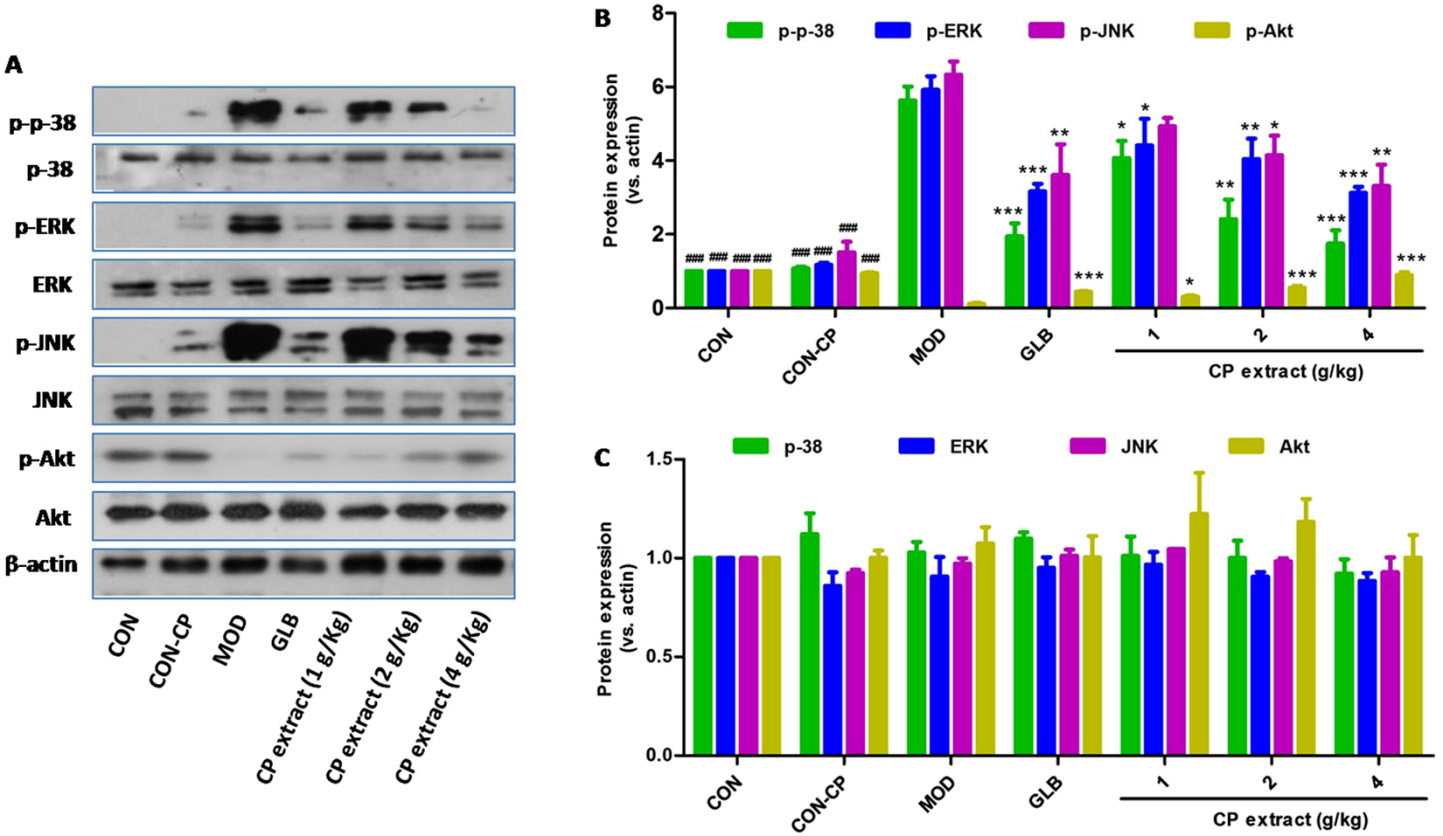
CP extract suppresses MAPK signals and activates Akt signal in pancrease tissues of diabetic mice. The type 2 diabetic mice were induced by feeding with high-fat diet for 4 weeks and then injecting intraperitoneally with 25 mg/kg STZ for 3 days consecutively. The diabetic mice with consecutive 7-day hyperglycemia (11 mmol/L or greater) were selected for the experiment and then CP extract or glibenclamide were administered to mice for consecutive 5 weeks. At the end of experiment, mice were sacrificed. The samples of pancrease tissues were collected. Protein expressions of p-p38, p38, p-ERK, ERK, p-JNK, JNK, p-Akt and Akt were determined in pancreas tissues using Western blot. (**A**) Representative image of Western blot; (**B**) Protein expressions of p-p38, p-ERK, p-JNK and p-Akt in pancreas tissues. (**C**) Protein expressions of p38, ERK, JNK, and Akt in pancreas tissues. All data are presented as means ± SEM (n = 8). ^#^ *p* < 0.05, ^##^ *p* < 0.01 and ^###^ *p* < 0.001, diabetic model group compared with non-diabetic groups; ^*^ *p* < 0.05, ^**^ *p* < 0.01 and ^***^ *p* < 0.001, compared with diabetic model group. CON: non-diabetic control group; CON-CP: CP extract-treated non-diabetic control group; MOD: diabetic model group; GLB: glibenclamide-treated diabetic group.

To identify whether Akt and mitogen-activated protein kinases are also involved in CP extract-induced pancreatic β cell protection, Western blot analysis was performed. As shown in Fig. 5, STZ and high fat induction resulted in drastic increases of ERK, JNK and p38 phosphorylation and a drastic decrease of Akt phosphorylation in pancreatic tissues of diabetic mice *vs*. non-diabetic mice, but those could significantly be reversed by CP extract or glibenclamide treatment, suggesting CP extract protects against pancreatic β cell apoptosis by modulating MAPK and Akt pathways.

### CP extract suppresses pancreatic β cell apoptosis *in vitro* by modulating MAPK and Akt pathways

To further explore the mechanism of CP extract-induced pancreatic β cell protection, we developed an *in vitro* apoptosis model of pancreatic β cells by stimulating NIT-1 cells (a pancreatic β cell line) with STZ. After STZ treatment, the cells exhibited a high apoptosis rate and 32 μM STZ was selected as the working concentration, which resulted in 30.9% cell death (Supplementary Fig. 3A). As well, cells were exposed to different concentrations of CP extract (0, 1, 25, 50, 100, 200, 500 μg/mL) for 24 h and exhibited no significant cytotoxicity to NIT-1 cells (Supplementary Fig. 3B). Then, the dose-response assay of CP extract was performed on STZ-induced NIT-1 cells.

**Figure 6:**
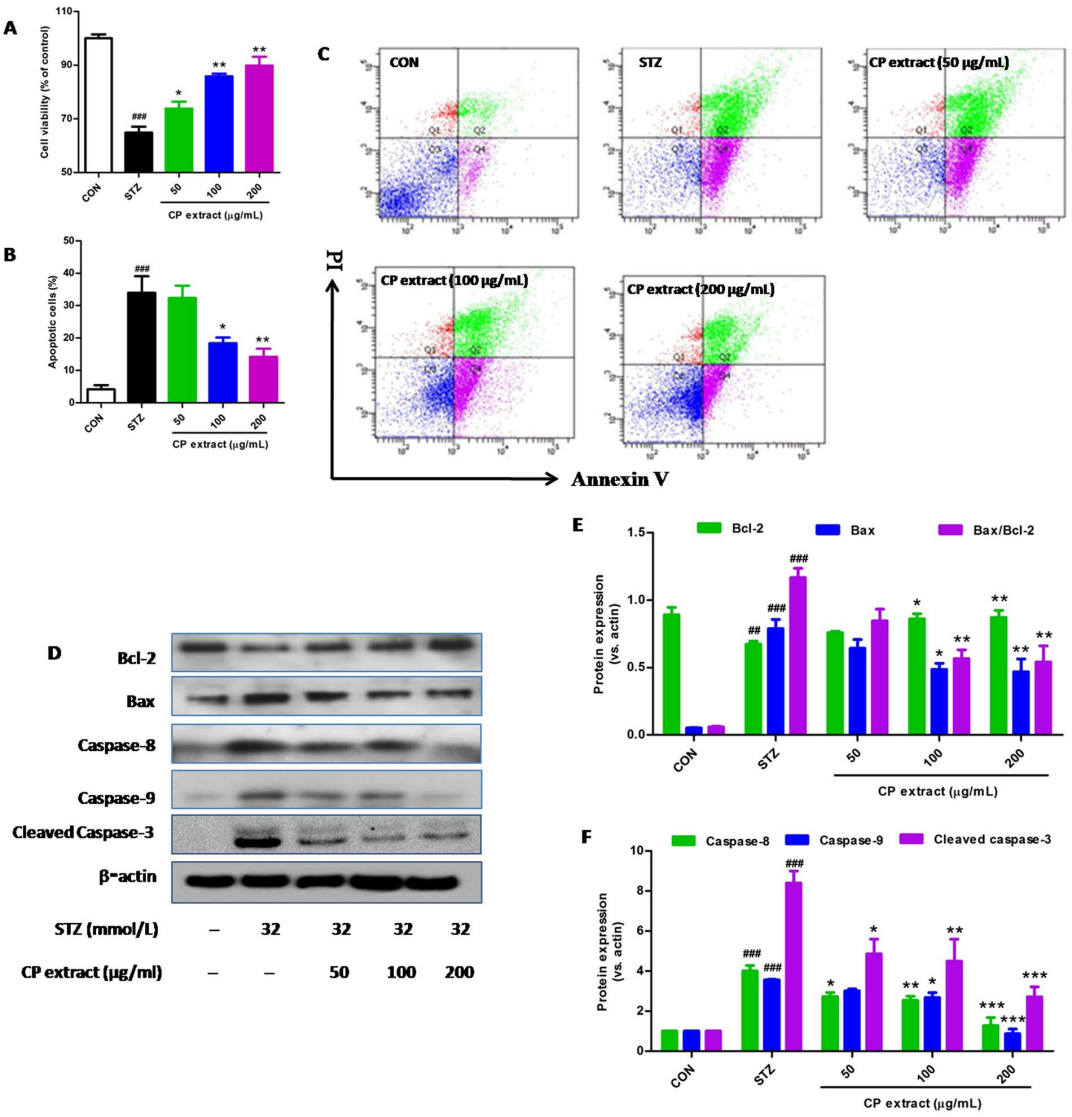
CP extract prevents STZ-induced pancreatic β cell apoptosis in NIT-1 cells. NIT-1 cells were preincubated with CP extract (50, 100 and 200 μg/mL) for 2 h before 32 mM STZ treated for 24 h. **(A)** Cell viability was assessed by MTT assay. (**B** and **C**) The apoptotic cells were stained with annexin V-FITC and PI, and then detected by flow cytometry. (**D**–**F**) Cells were lysed and then the expression of Bcl-2, Bax, caspase-8, Caspase-9 and cleaved caspase-3 were measured using Western blot. Data were expressed as mean ± SEM from three independent experiments. ^###^ *p* < 0.001, compared with untreated control group; *p < 0.05, compared with STZ-treated group.

**Figure 7:**
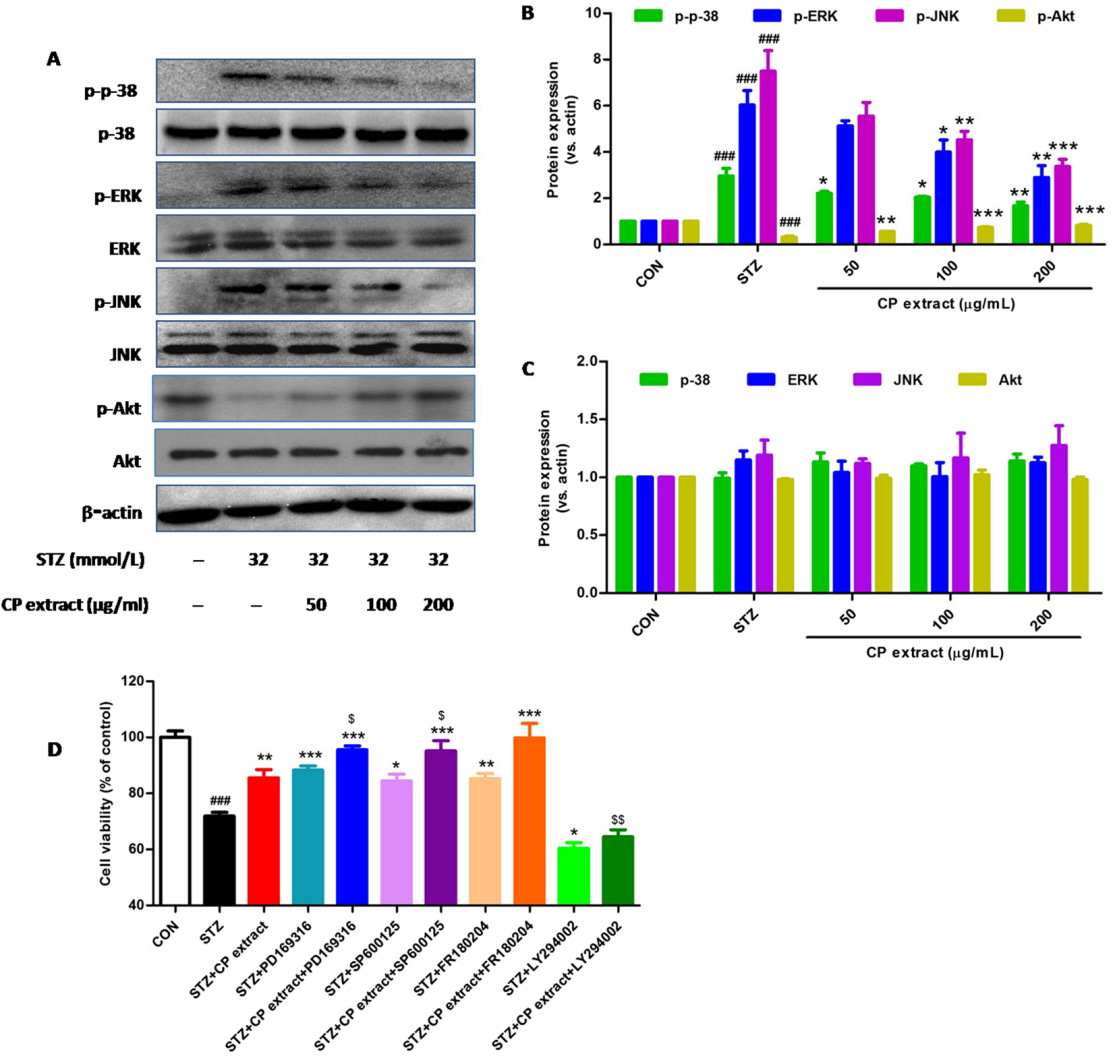
CP extract suppresses MAPK signals and activates Akt signal in STZ-induced NIT-1 cells. NIT-1 cellswere preincubated with CP extract (50, 100 and 200 μg/mL) for 2 h before 32 mM STZ treated for 24 h. Cells were lysed and then the expression of proteins in MAPK and Akt signaling pathways were measured using Western blot. (**A**) Representative image of Western blot; (**B** and **C**) Protein expressions of p-p38, p38, p-ERK, ERK, p-JNK, JNK, p-Akt and Akt in NIT-1 cells. (**D**) NIT-1 cells were preincubated with CP extract (100 μg/mL) or PI3K inhibitor (LY294002, 1.25 μM) or ERK inhibitor (FR180204, 1.25 μM) or p-38 inhibitor (PD169316, 1.25 μM) or JNK inhibitor (SP600125, 1.25 μM) or combnation of both CP extract and LY294002 or FR180204 or PD169316 or SP600125 for 2 h before 32 mM STZ treated for 24 h, then the cell viability was assessed by MTT assay. Data were expressed as mean ± SEM from three independent experiments. ^###^ *p* < 0.001, compared with untreated control group; *p < 0.05, compared with STZ-treated group; ^$^ *p* < 0.05 and ^$$^ *p* < 0.01, compared with STZ + CP extract group using Student’s t-test.

As shown in Fig. 6A, pretreatment with CP extract (50–200 μg/mL) could significantly inhibit cell death induced by STZ in a dose-dependent manner. To quantitatively examine the effect of CP extract on STZ-induced NIT-1 cell apoptosis, the percentage of apoptotic cells was detected by Annexin V/PI double staining methods. As shown in Fig. 6B,C, the percentage of apoptotic cells was significant increased after 32 μM STZ treatment, and pretreatment with CP extract (50, 100, 200 μg/mL) significantly reduced the percentage of apoptotic cells in a dose-dependent manner in comparison with the cells treated with STZ alone. Activation of caspases is responsible for apoptosis execution^11^. As shown in Fig. 6D–F, the expression of caspase-8, caspase-9, cleaved caspase-3 and Bax/Bcl-2 ratio was markedly up-regulated after STZ treatment. In contrast, pretreatment with CP extract significantly inhibited the up-regulation of caspase-8, caspase-9, cleaved caspase-3 and Bax/Bcl-2 ratio. To confirm Akt and MAPK kinases are also involved in CP extract-induced pancreatic β cell protection, Western blot analysis was performed. As shown in Fig. 7A–C, treatment with STZ resulted in a significant increase of phosphorylated p38, ERK and JNK and a drastic decrease of phosphorylated Akt, but have no change on total p38, ERK, JNK and Akt. As expected, increased phosphorylation of p38, JNK and ERK, as well as decreased phosphorylation of Akt were significantly rescued by CP extract with a positive dose-dependent relationship. To furter confirm the role of CP extract in regulation of MAPK and Akt signaling pathways, NIT-1 cells were treated with inhibitor of PI3K (LY294002) and/or ERK (FR180204) and/or p-38 (PD169316) and/or JNK (SP600125), respectively. As expected, the cell viability of STZ-treated NIT-1 cells treated with CP extreat in combination of PD169316 or FR180204 or SP600125, were much higher than that of CP extreat treatment alone, whereas the cell viability of STZ-treated NIT-1 cells treated with CP extreat in combination of LY294002 was much lower than that of CP extreat treatment alone (Fig. 7D). These data demonstrate that the protective effect of CP extract on STZ-induced apoptosis in NIT-1 cells is mediated by modulating MAPK and Akt signaling pathways.

### CP extract reduces diabetes-related complications in diabetic mice

**Figure 8:**
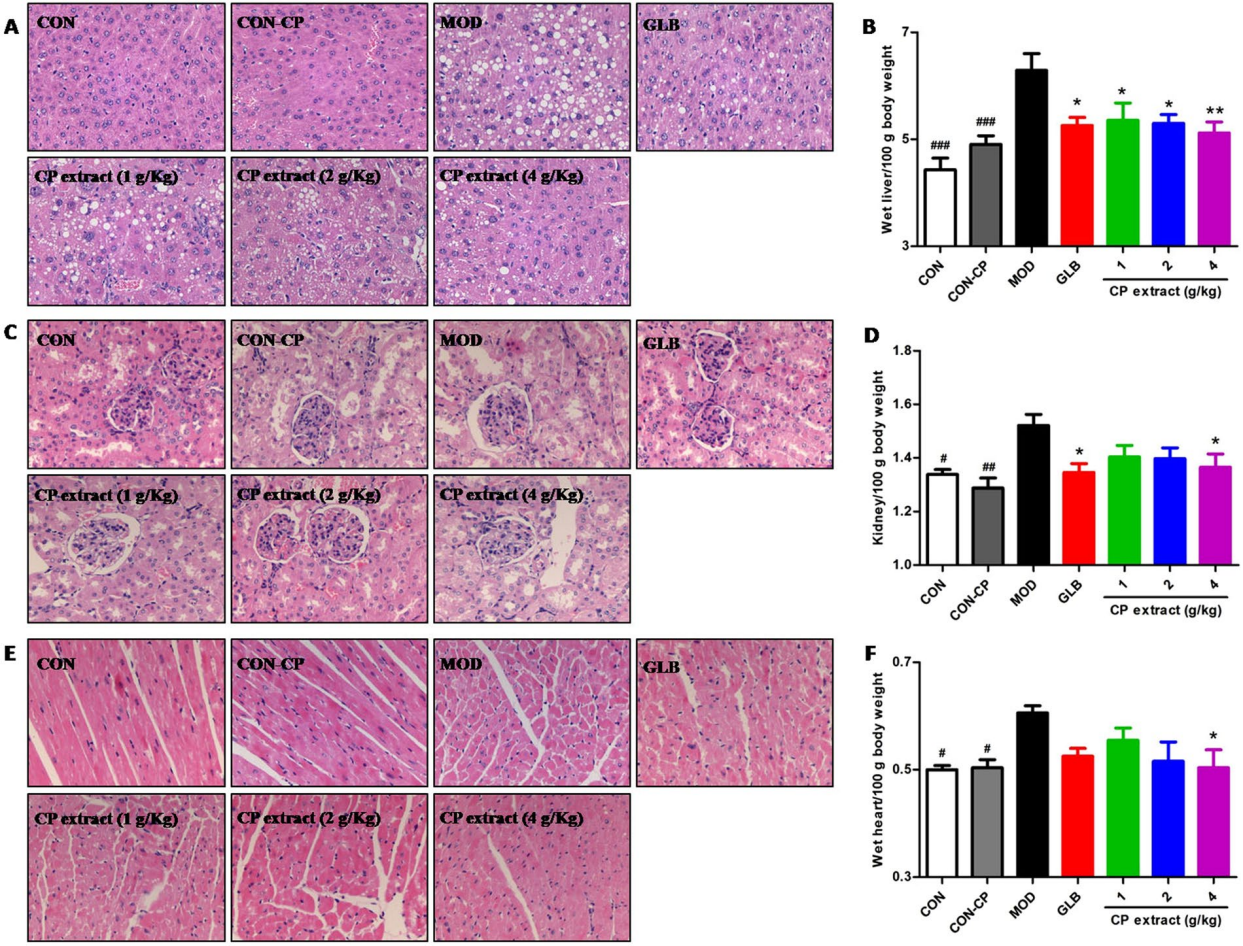
CP extract reduces chronic hyperglycemia-induced damages in liver, kidney and heart of diabetic mice. The type 2 diabetic mice were induced by feeding with high-fat diet for 4 weeks and then injecting intraperitoneally with 25 mg/kg STZ for 3 days consecutively. The diabetic mice with consecutive 7-day hyperglycemia (11 mmol/L or greater) were selected for the experiment and then CP extract or glibenclamide were administered to mice for consecutive 5 weeks. At the end of experiment, mice were sacrificed. The samples of liver, kidney and heart tissues were collected. Histopathological examination of liver (**A**), kidney (**C**) and heart tissues (**E**) (H & E staining) (magnification, 100×); Relative weight of wet liver (**B**), kidney (**D**) and heart tissues (**F**). All data are presented as means ± SEM (n = 8). ^#^ *p* < 0.05, ^##^ *p* < 0.01 and ^###^ *p* < 0.001, diabetic model group compared with non-diabetic groups; ^*^ *p* < 0.05 and ^**^ *p* < 0.01, compared with diabetic model group. CON: non-diabetic control group; CON-CP: CP extract-treated non-diabetic control group; MOD: diabetic model group; GLB: glibenclamide-treated diabetic group.

**Figure 9:**
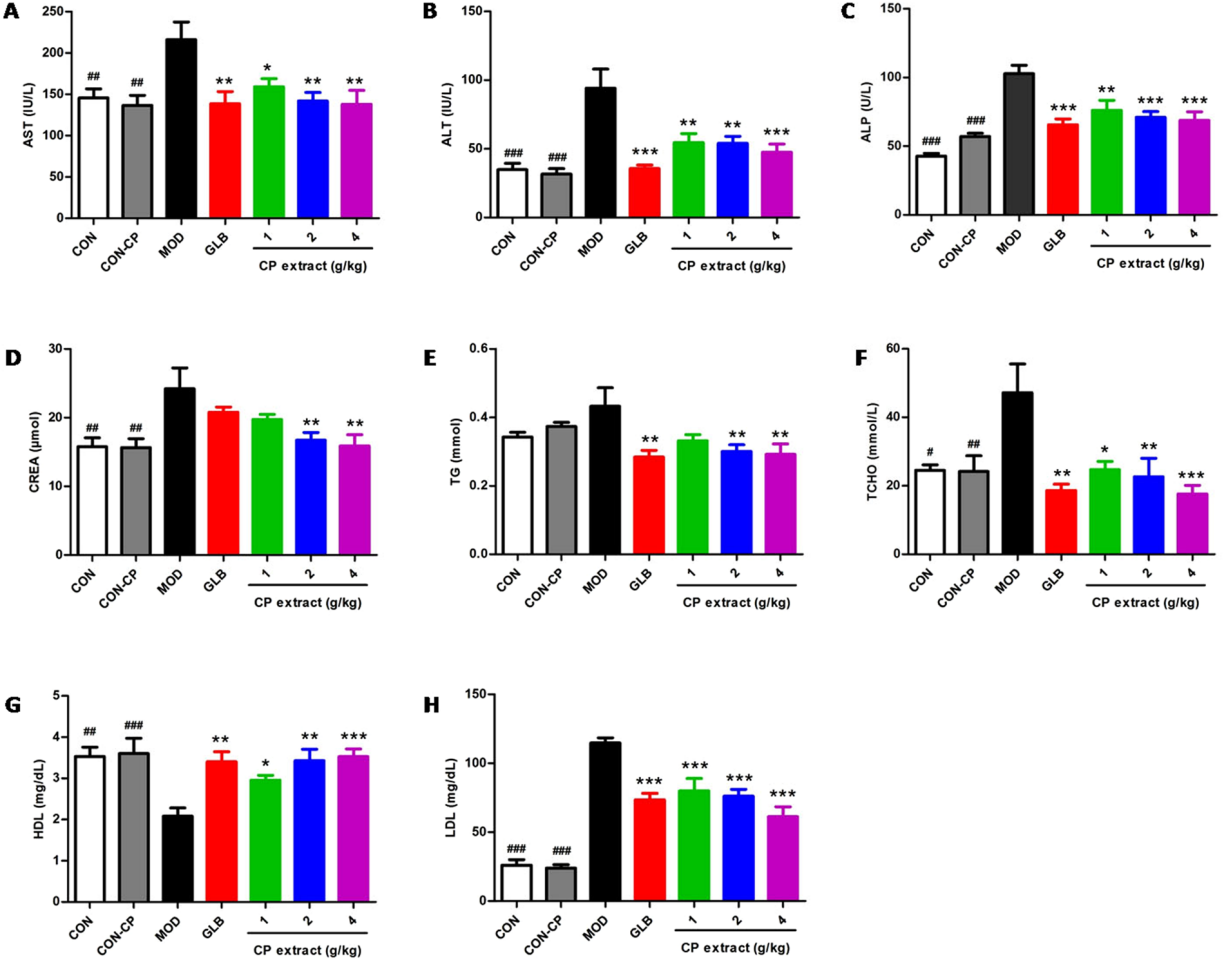
The effects of CP extract on the blood biochemical changes of diabetic mice. The type 2 diabetic mice were induced by feeding with high-fat diet for 4 weeks and then injecting intraperitoneally with 25 mg/kg STZ for 3 days consecutively. The diabetic mice with consecutive 7-day hyperglycemia (11 mmol/L or greater) were selected for the experiment and then CP extract or glibenclamide were administered to mice for consecutive 5 weeks. At the end of experiment, mice were sacrificed. The samples of blood were collected and relevant blood biochemicals in serum were measured by using corresponding commercially available kits. Blood (**A**) ALT, (**B**) AST, (**C**) ALP, (**D**) CREA, (**E**)TG, (**F**) TCHO, (**G**) LDL and (**H**) HDL levels. All data are presented as means ± SEM (n = 8). ^#^ *p* < 0.05, ^##^ *p* < 0.01 and ^###^ *p* < 0.001, diabetic model group compared with non-diabetic groups; ^*^ *p* < 0.05, ^**^ *p* < 0.01 and ^***^ *p* < 0.001, compared with diabetic model group. CON: non-diabetic control group; CON-CP: CP extract-treated non-diabetic control group; MOD: diabetic model group; GLB: glibenclamide-treated diabetic group.

Chronic hyperglycemia can cause many major health complications, such as renal disease, hepatopathy and heart disease. We also detected the protective effects of CP extract on the liver, kidney and heart of diabetic mice. As shown in Fig. 8, histological examination displayed amount of large and small vesicles of fat accumulating within hepatocytes, a typical pathological feature of hepatic steatosis, in the liver sections (Fig. 8A); and a thicker glomerular basement membrane in the kidney section (Fig. 8C), as well as observable cardiomyocyte hypertrophy in heart section (Fig. 8E,F) of diabetic mice, in comparison with samples from non-diabetic mice. After supplementation of CP extract or glibenclamide, the hepatic steatosis, glomerular basement membrane thicken and cardiac hypertrophy were greatly improved. In parallel, the data showed the heart/body weight ratio of mice in diabetic mice was higher than that of non-diabetic mice, and this rise was largely suppressed after CP extract or glibenclamide adminstration (*P* < 0.05)(Fig. 8F). Subsequently, we detected the blood biochemical changes of mice and showed that the levels of AST, ALT, CREA, TG, TCHO and LDL/VLDL in the serum of diabetic mice were markedly elevated, whereas HDL in the serum of diabetic mice were significantly decreased *vs*. those in mice of non-diabetic mice. And those elevations or decline were significantly rescued by CP extract treatment in a dose-dependent manner (Fig. 9). Collectively, these data displayed a potent beneficial effect of CP extract in prevention of diabetic complications such as hepatic steatosis, nephropathy and cardiac hypertrophy.

## Discussion

Current research in diabetes reveals that insufficient insulin secretion due to β cell loss is the common and major component in the pathogenesis of diabetes, and a significant reduction in pancreatic β cell populations is the primary mechanisms for β cell loss^1, 12^. Inhibiting pancreatic β cell apoptosis thus represent a potential strategy to combat diabetes and its complications. In the present study, we explored the protective role of *C. paliurus* tea leaves on pancreatic β cells. Our data consistently revealed that CP extract exhibit a potential anti-hyperglycemic effect in diabetic mice and has obvious protective effects against the apoptosis of pancreatic β cells, in agreement with previous studies demonstrating that *C. paliurus* is benefcial to diabetics. Our data support a clear antidiabetic effect of *C. paliurus* tea leaves through regulating β cell preservation by protection against pancreatic β cell apoptosis.

To characterize the antidiabetic effect of CP extract *in vivo*, high-fat diet with low dose of STZ-induced type 2 diabetic mice were enrolled in the study. It is well known that high-fat diet would lead to insulin resistance and/or glucose intolerance, and the subsequent administration of multiple low dose of STZ, a *β* cell toxin, would result in a severe reduction in functional *β* cell mass^13–15^. Based on this model, we oral administration of CP extract to the diabetic mice and accessed its therapeutic effect. Our findings demonstrate the body loss, food intake and serum plasma glucose levels were significantly decreased in the CP extract-treated groups, while the wet pancreas/body weight ratio, serum plasma insulin levels, β-cell area and insulin-producing β cells were markedly increased, which are in line with our findings that TUNEL-positive cells, the expression of caspase-8, caspase-9 and cleaved caspase-3 in the islets of CP extract -treated group were lower than those of diabetic model group. NIT-1, a pancreatic cell line, showed many characteristics and ultrastructural features of normal differentiated mouse pancreatic β cells^16^. Using this cell line, we also developed *in vitro* apoptosis model of pancreatic β cells by challenging with STZ. Our results showed that CP extract could significantly reduce the percentage of apoptotic cells, as well as inhibit the up-regulation of cleaved caspase-3 and caspase-9 induced by STZ in a dose-dependent manner. Collectively, those data reveal potential antidiabetic effect of *C. paliurus* tea through protecting against pancreatic β cell apoptosis.

Kinase signaling pathways are regulated in response to various extracellular physical (e.g., UV radiation, and temperature) and chemical (many agents) stimuli. They can be involved, depending on cell type, in the regulation of many cellular processes such as proliferation, differentiation, inflammatory response, autophagy, senescence, and also in apoptosis. Šrámek J. *et al*. reviewed that Akt kinases and kinases in MAPK superfamily such as ERKs, JNK and p38, play important roles in regulating apoptosis of pancreatic β cells^17^. We further explored whether the Akt, p38, ERK, and JNK signaling pathways in pancreas islets of diabetic mice can be influenced by the treatment of CP extract. Our data showed that treatment with CP extract significantly suppressed ERK, JNK and p38 phosphorylation, and increased Akt phosphorylation in pancreatic tissues of diabetic mice. In line with results from *in vivo*, treatment of CP extract is effective to suppress the increased ERK, JNK and p38 phosphorylation, and elevate the decreased Akt phosphorylation in NIT-1 cells, which are induced by STZ, in a dose-dependent manner. *In vitro* study also showed that ERK, JNK and p38 inhibitors could greatly enhance, whereas PI3K inhibitor could significantly weaken the protective effect of CP extract against STZ-induced apoptosis. These findings suggest that CP extract protects pancreatic β cells from apoptosis *via* affecting MAPK and Akt signaling pathways.

It is well known that chronic hyperglycemia may lead to multi-organ damage through activating different mechanisms such as an increased polyol pathway, advanced-glycation end product formation, activation of Protein Kinase C and hexosamine pathway, resulting in overproduction of reactive oxygen species to impair various tissues^18^. These associated lesions of diabetes are termed as diabetic complications. Based on the tissue affected, these complications are named accordingly as diabetic hepatopathy, diabetic nephropathy, and diabetic cardiomyopathy, etc. Hepatic steatosis is a typical pathological feature of diabetic hepatopathy with abnormalities in lipid metabolism^19^. The alterations in the plasma lipid profile (elevated triglycerides, total cholesterol, LDL/VLDL; and reduced HDL) and liver injury markers (elevated AST, ALT and ALP) of the diabetic mice in this study are in agreement with the alterations of high-fat diet with low dose of STZ induced type 2 diabetic mice reported by other researchers^20, 21^. The decrease in the pathological changes on hepatic steatosis and levels of AST, ALT, TG, TCHO and LDL/VLDL in plasma, as well as an increase of plasma HDL in the diabetic mice fed CP extract-supplemented diets, in comparison with the diabetic model group indicates a potent hepatoprotective effect of CP extract. Diabetic nephropathy is another complication of diabetes, which begins with glomerular hyperfiltration caused by hyperglycemia, and further results in glomerular hypertrophy and glomerular basement membrane thickening^22^. Our data showed CP extract is also effective to improve the nephropathy of diabetic mice evidenced by decreasing glomerular basement membrane thickening, reducing the wet kidney/body weight ratio and lowering the level of plasma CREA. In the myocardium, chronic hyperglycemia can cause cardiac hypertrophy directly^23^. As well, compared to diabetic model group, the decrease in both the pathological changes on cardiac hypertrophy and wet heart/body weight ratio in CP extract-treated diabetic mice also indicates cardioprotective potential of CP extract. Collectively, these data suggest that CP extract plays a beneficial role in protection against diabetes-related complications hepatic steatosis, nephropathy and cardiac hypertrophy or inhibit their development.

In summary, CP extract exhibit a potential anti-hyperglycemic effect in high-fat diet with low dose of STZ-induced type 2 diabetic mice, and protecting against diabetes-related complications. Those effects are associated with protecting pancreatic β cells from apoptosis *via* modulating MAPK and Akt signaling pathways.

## Materials and Methods

### Preparation of CP extract

The leaves of *Cyclocarya paliurus* (Batal.) Iljinskaja were collected in the garden of Nanjing Forestry University, China (Nanjing, Jiangsu, China) in March, 2015. The sample was authenticated by Prof. Hu biao Chen of School of Chinese Medicine, Hong Kong Baptist University, and voucher specimens (No. CP20151201) was stored in our Research Laboratory, School of Chinese Medicine, Hong Kong Baptist University, Hong Kong. For preparation of the extract of *C. paliurus* leaves (CP extract), the air-dried leaves of *C. paliurus* (5 Kg) were boiled with water (60 L water for the first time and 50 L water for the second time) for twice (boiled 2 h for the first time and boiled 1 h for the second time). The extract was then concentrated and dried under reduced pressure to yield the crude extract (830 g). The residue was stored at 4 °C, and the desired dose was reconstituted in 0.5% CMC-Na solution.

### Phytochemical analysis

The powder of CP extract was extracted twice for 30 min each time by ultrasound in 70% methanol (1:10, w/v). After being filtered and combined, the filtrate was passed through a 0.22 μm membrane before using for Ultra Performance Liquid Chromatography (UPLC) analysis. The chromatographic analysis was performed on a Waters ACQUITY UPLC system equipped with an auto sampler, binary gradient pump, and PDA detector, connected to an Empowder ChemStation software (Waters, MA, USA). The system was operated at 30 °C and a water ACQUITY UPLC HSS T3 column (150 × 2.1 mm, 1.7 μm) was used. The injection volume was 5 μL and the mobile phase flow rate was 0.4 mL/min. Solvents that constituted the mobile phase were (A) 0.2% aqueous acetic acid and (B) acetonitrile. The elution conditions were as follows: 0–5 min, linear gradient 2–5% B; 5–10 min, linear gradient 5–10% B; 10–15 min, linear gradient 10–25% B; 15–25 min, linear gradient 25–40% B; 25–28 min, linear gradient 40–90% B. Peaks were detected at 254 nm.

### Animals and experimental procedures

Male C57/BL6J mice of 8-week-old were purchased from the Laboratory Animal Services Center, The Chinese University of Hong Kong. The animals were fed a standard rodent diet with free access to water, and were kept in rooms maintained at 22 ± 1 °C with a 12 h light/dark cycle following international recommendations. All experimental protocols were approved by the Animal Ethics Committees of Hong Kong Baptist University, in accordance with “Institutional Guidelines and Animal Ordinance” (Department of Health, Hong Kong Special Administrative Region) (Registration No. LIUYE/15-16/01-CLNC).

The inducement of diabetic animals was carried out according to a previous reported procedure^24^. In brief, the animals were fed with high-fat diet (Adjusted Calories Diet (42% from fat) (No. 881372)) (Harlan Laboratories, Inc, IN Indianapolis, USA) for 4 weeks before consecutive intraperitoneal (i.p) injection with 25 mg/kg streptozotocin (STZ, Sigma-Aldrich, St. Louis, MO, USA) for 3 days. An equal volume of vehicle was injected into the control mice. The blood glucose levels in serum were determined by a glucometer (OMRON (China) Co., Ltd, Beijing, China). The mice with fasting glucose level higher than 11 mmol/L were considered as diabetic mice, and the diabetic mice with consecutive 7-day hyperglycemia (11 mmol/L or greater) were used for the experiment. The diabetic mice were randomly divided into five groups: diabetic group, glibenclamide-treated diabetic group, low dose of CP extract-treated diabetic group, medium dose of CP extract-treated diabetic group, and high dose of CP extract-treated diabetic group. The untreated mice were matched in age and divided into two groups: vehicle control group and CP extract-treated control group. For the treatment, CP extract was orally administrated to diabetic mice at doses of 1, 2, 4 g/kg/day according to the result of preliminary experiment. Glibenclamide (Sigma-Aldrich, St. Louis, MO, USA) was used as a reference positive agent and it was given at 15 mg/kg/day according to literature^25^. CP extract-treated control group was given CP extract at a dose of 2 g/kg/day, whereas the vehicle control group and diabetic model group were orally fed with the same volume of 0.5% CMC-Na solution instead of CP extract or glibenclamide. Both CP extract and glibenclamide were freshly dissolved in 0.5% CMC-Na solution respectively and orally administrated to the mice for 5 weeks after the onset of treatment. Body weight, food consumption and blood glucose levels in each mice were monitored every 7 days. Blood samples were obtained from the tail vein of the mice and blood glucose levels in serum were determined by a glucometer (OMRON (China) Co., Ltd, Beijing, China).

### Glucose tolerance test (GTT)

The glucose tolerance test (GTT) was carried out after 4 weeks of treatment. Mice were oral administrated with 2 g/kg body weight of D-glucose (Sigma-Aldrich, St. Louis, MO, USA) after 16 h fast. Blood samples were taken from the tail vein of the mice at 0, 30, 60, 90 and 120 min after glucose administration, respectively. The GTT was carried out on awake mice without anesthetization. The glucose levels in serum were also determined by a glucometer (OMRON (China) Co., Ltd, Beijing, China).

### Blood biochemical analysis and tissue preparation

At the end of the experiment, mice were sacrificed and blood samples were obtained from the abdominal vein with a microsyringe. Serum was separated at 3000 rpm for 15 min. Blood insulin, high-density lipoprotein cholesterol (HDL) and low-density and very low lipoprotein cholesterol (LDL/VLDL) levels, AST, ALT, ALP, TG, TCHO and CREA were measured in a biochemical analyzer (Hitachi 902 Automatic Analyzer; Hitachi, Japan) with adapted reagents from Shanghai Kehua Bio-engineering Co., Ltd (Shanghai, China). As well, pancreas, liver, kidney and heart were immediately separated and collected.

### Histological analysis

Pancreas, liver, kidney and heart tissues were post-fixed in 4% PFA for 24 hr and sectioned after embedding in paraffin. The sections were prepared and stained with H&E using standard procedures. All slides in the current study were examined under a light microscope (Olympus BX51, Olympus Co., Tokyo, Japan) and photographed at high resolution with a digital camera (Olympus C-5050, Olympus Co., Tokyo, Japan) unless otherwise noted.

### Immunoblotting

Immunoblotting was performed as previously described^26^. Briefly, proteins were extracted from the pancreas tissues and NIT-1 cells in the presence of a cocktail of protease inhibitors and phenylmethylsulfonyl fluoride (PMSF) (Roche Applied Science, Branford, CT, USA), resolved and separated by 12–15% SDS-PAGE, and transferred to PVDF membranes. The membranes were then probed with the primary antibodies caspase-8, caspase-9, cleaved caspase-3, Bax, Bcl-2, ERK, JNK, p38, p-ERK, p-JNK,p-p38, Akt, p-Akt and β-actin (Cell Signaling Technology, Inc. Beverly, MA, USA), followed by incubation with the corresponding horseradish peroxidase-conjugated secondary antibodies. The protein bands were visualized with enhanced chemiluminescence reagents (GE Healthcare Bio-Sciences Corp, Piscataway, NJ, USA), detected using the ECL system, and quantifed using NIH ImageJ sofware (National Institutes of Health, Bethesda, Maryland, USA).

### Immunohistochemistry and immunofluorescence

*In Situ* Cell Apoptosis Detection Kit (Millipore, Danvers, MA, USA) was used to evaluate the β cell apoptosis in pancreas following the manufacturer’s instructions. Briefly, the sections were heated at 60 °C for 2 h, followed by deparaffinization and rehydration. Then the sections of pancreas were treated with proteinase K for 5 min at room temperature, and washed twice with PBS. After incubation with 60 μl TUNEL reaction mixture (Label Solution and Enzyme Solution; 5:1) following the manufacturer’s recommendations, for 60 min at 37 °C without light, then the slides sections were incubated with the prediluted anti-digoxin antibody, followed with ABC for 30 min. Apoptotic cells were detected by incubation with the 3,3-diaminobenzidine (DAB) chromogen.

For immunofluorescence staining, sections were permeabilized in PBS with 0.1% Triton X-100 for 10 min after deparaffinized and rehydrating. Then the blocking was performed using PBS with 5% normal goat serum for 1 h at room temperature, followed by incubation with rabbit anti-insulin antibody and mouse anti-cleaved caspase-3 antibody (Cell Signaling Technology, Danvers, MA, USA) diluted 1:100 at 4 °C overnight. Three consecutive washes with PBS for 5 min each were followed by sequential incubation with Alexa Fluor 595 and 488 goat anti-rabbit and anti-mouse IgG secondary antibodies (1:200) at room temperature for 1 h. The slides were washed three times with PBS and mounted using an anti-fade mounting medium containing 4′,6-diamidino-2-phenylindole (DAPI) (Vector Laboratories, Burlingame, CA, USA). Images were captured under a fluorescence microscope (Carl Zeiss AG; Oberkochen, Germany).

### Cell Culture

NIT-1 cells were obtained from American Type Tissue Culture Collection (passage 40–60) and cultured under an atmosphere of 5% CO_2_ and 95% O_2_ at 37 °C in RPMI 1640 medium (Gibco Chemical, USA) containing 10% (v/v) fetal bovine serum (FBS), 11.1 mM glucose, 100 U/mL penicillin, and 0.1 mg/mL streptomycin.

### Cell viability assay and flow cytometry

Cell viability assay was determined by MTT method, the live cell number was determined using a microplate reader based on absorbance values. Relative cell apoptosis was measured by flow cytometry (Becton Dickinson, San Jose, CA, USA)using Annexin V-FITC Apoptosis Detection Kit (eBioscience, Bender MedSystems, GmbH, Vienna, Austria).

### Statistical analysis

All data were analyzed with GraphPad PRISM 5.0 (GraphPad Software Inc., San Diego, CA, USA) sofware using one-way ANOVA, followed by Duncan’s multiple range tests unless otherwise noted. All quantitative values are expressed as the mean ± SEM. Differences were deemed signifcant when *P* < 0.05.

## Electronic supplementary material

Supplementary Information
