# Author Correction: *Cyclocarya paliurus* tea leaves enhances pancreatic β cell preservation through inhibition of apoptosis

**DOI:** 10.1038/s41598-020-74829-9

**Published:** 2020-11-04

**Authors:** Hai-tao Xiao, Bo Wen, Zi-wan Ning, Li-xiang Zhai, Cheng-hui Liao, Cheng-yuan Lin, Huai-xue Mu, Zhao-xiang Bian

**Affiliations:** 1 School of Chinese Medicine, Hong Kong Baptist University, Kowloon Tong, Kowloon, Hong Kong; 2 Shenzhen Research Institute and Continuing Education, Hong Kong Baptist University, Shenzhen, China

Correction to: *Scientific Reports* https://doi.org/10.1038/s41598-017-09641-z, published online 22 August 2017

This Article contains an error in Figure 9 where the y-axis label in panel (F) is incorrect. The correct Figure 9 appears below as Figure 1.

**Figure 1:**
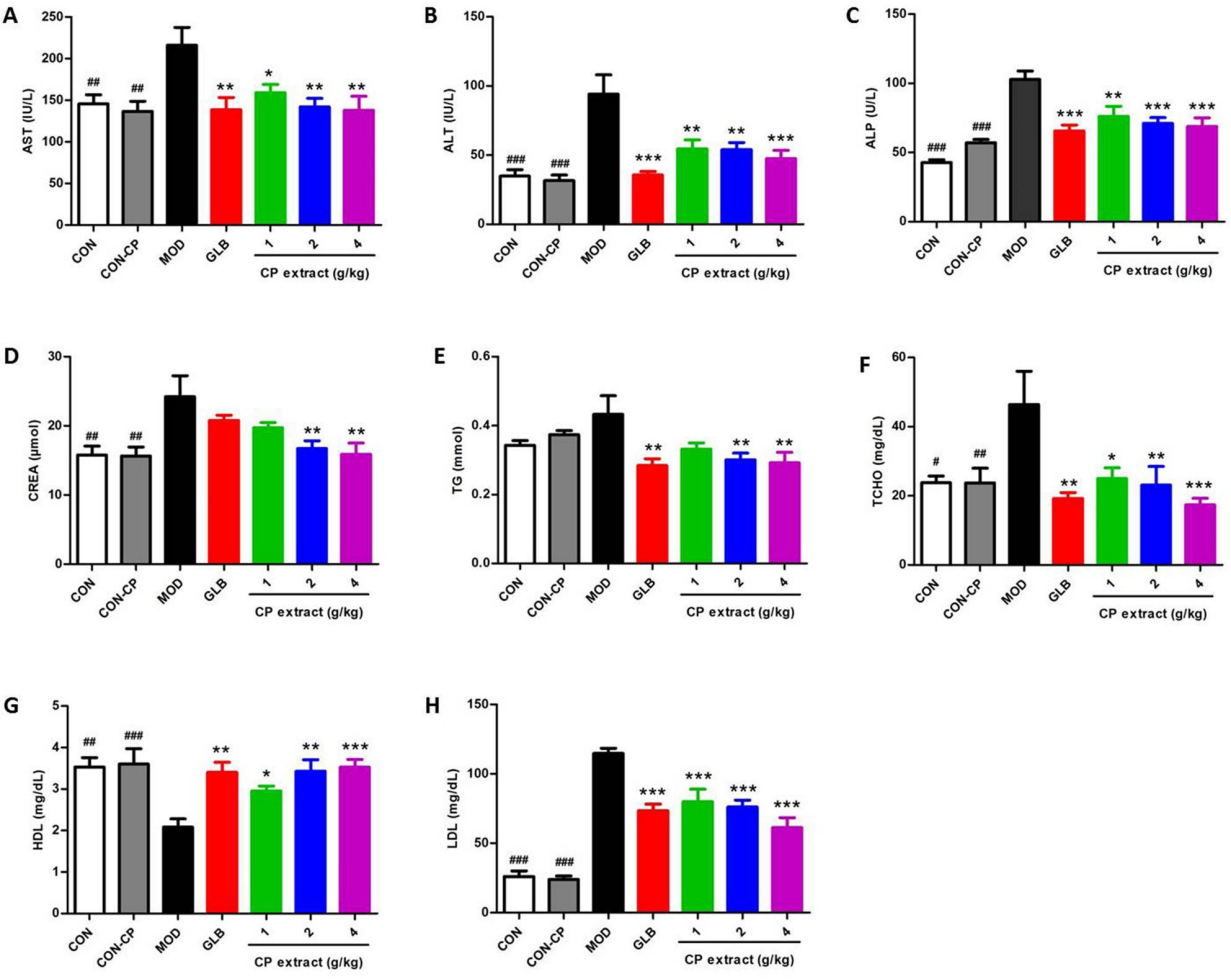
The effects of CP extract on the blood biochemical changes of diabetic mice. The type 2 diabetic mice were induced by feeding with high-fat diet for 4 weeks and then injecting intraperitoneally with 25 mg/kg STZ for 3 days consecutively. The diabetic mice with consecutive 7-day hyperglycemia (11 mmol/L or greater) were selected for the experiment and then CP extract or glibenclamide were administered to mice for consecutive 5 weeks. At the end of experiment, mice were sacrificed. The samples of blood were collected and relevant blood biochemicals in serum were measured by using corresponding commercially available kits. Blood (**A**) ALT, (**B**) AST, (**C**) ALP, (**D**) CREA, (**E**) TG, (**F**) TCHO, (**G**) LDL and (**H**) HDL levels. All data are presented as means ± SEM (n = 8). ^#^ *p* < 0.05, ^##^ *p* < 0.01 and ^###^ *p* < 0.001, diabetic model group compared with non-diabetic groups; ^*^ *p* < 0.05, ^**^ *p* < 0.01 and ^***^ *p* < 0.001, compared with diabetic model group. CON: non-diabetic control group; CON-CP: CP extract-treated non-diabetic control group; MOD: diabetic model group; GLB: glibenclamide-treated diabetic group.

