# Supplementary material for: Cyclocarya paliurus tea leaves enhances pancreatic β cell preservation through inhibition of apoptosis
Source: Sci Rep. 2017 Aug 22;7:9155. doi: 10.1038/s41598-017-09641-z (PMC5567240; doi:10.1038/s41598-017-09641-z)
Supplement: Supplementary file 1 — Supplementary Information [file 41598_2017_9641_MOESM1_ESM.pdf]

Supplementary Information

***Cyclocarya paliurus* tea leaves enhances pancreatic β cell preservation through inhibition of apoptosis**

Hai-tao Xiao[1,2*], Bo Wen[1,2*], Zi-wan Ning[1], Li-xiang Zhai[1], Cheng-hui Liao[2], Cheng-yuan Lin[1,2], Huai-xue Mu[1,2], Zhao-xiang Bian[1,2**]

[1] School of Chinese Medicine, Hong Kong Baptist University, Kowloon Tong, Kowloon, Hong Kong

[2] Shenzhen Research Institute and Continuing Education, Hong Kong Baptist University, Shenzhen, China

[*]Equal contribution.

[**]**Correspondence:** Prof. Zhao-xiang Bian, School of Chinese Medicine, Hong Kong Baptist University, Kowloon Tong, Kowloon, Hong Kong， P. R. China; Fax: +852-3411-2929, Tel: +852-3411-2905; E-mail: bzxiang@hkbu.edu.hk

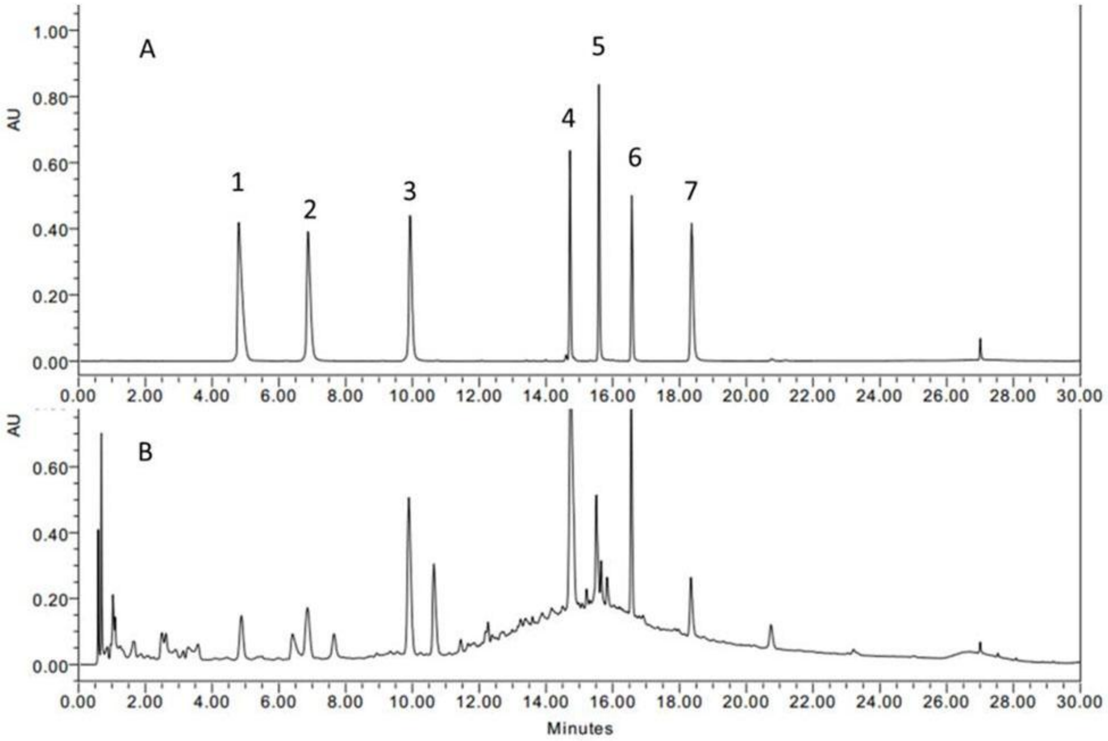

Fig S1 UPLC-PDA chromatogram of standard mixture (**A**) and CP extract (**B**). External standard: (**1**) 1-caffeoylquinic acid, (**2**) 5-caffeoylquinic acid, (**3**) chlorogenic acid, (**4**) isoquercitrin, (**5**) kaempferol-3-glucoside, (**6**) kaempferol 3-rhamnoside, and (**7**) quercetin.

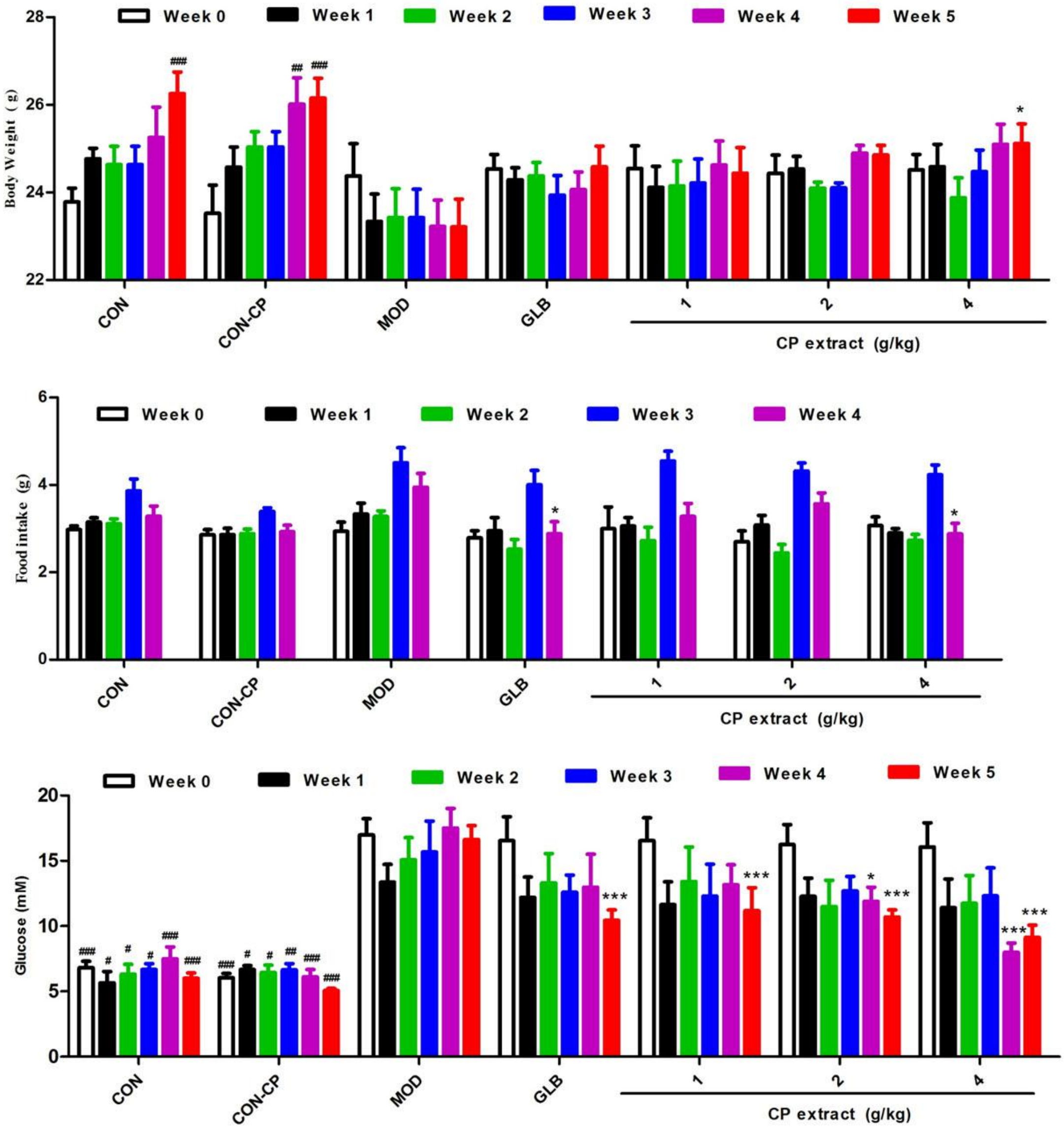

Fig S2 The dynamic changes of body weight, food intake and blood glucose levels of non-diabetic and diabetic mice after CP extract treatment. (**A**) The dynamic changes of body weight; (**B**) The dynamic changes of food intake; (**C**) The dynamic changes of blood glucose levels. The type 2 diabetic mice were induced by feeding with high-fat diet for 4 weeks and then injecting intraperitoneally with 25 mg/kg STZ for 3 days consecutively. The diabetic mice with consecutive 7-day hyperglycemia (11 mmol/L or greater) were selected for the experiment and then CP extract or glibenclamide were administered to mice for consecutive 5 weeks. During the experiment, the body weight, food intake and blood glucose levels of mice

were monitored weekly. The blood glucose levels in serum were determined by a glucose measuring kit (Biosino Bio-technology and Sicence, China) using the glucose oxidase method. All data are presented as means ± SEM (n = 8). $^{\#}p < 0.05$, $^{\#\#}p < 0.01$ and $^{\#\#\#}p < 0.001$, compared with non-diabetic groups; $^{*}p < 0.05$, $^{**}p < 0.01$ and $^{***}p < 0.001$, compared with diabetic model group. CON: non-diabetic control group; CON-CP: CP extract-treated non-diabetic control group; MOD: diabetic model group; GLB: glibenclamide-treated diabetic group.

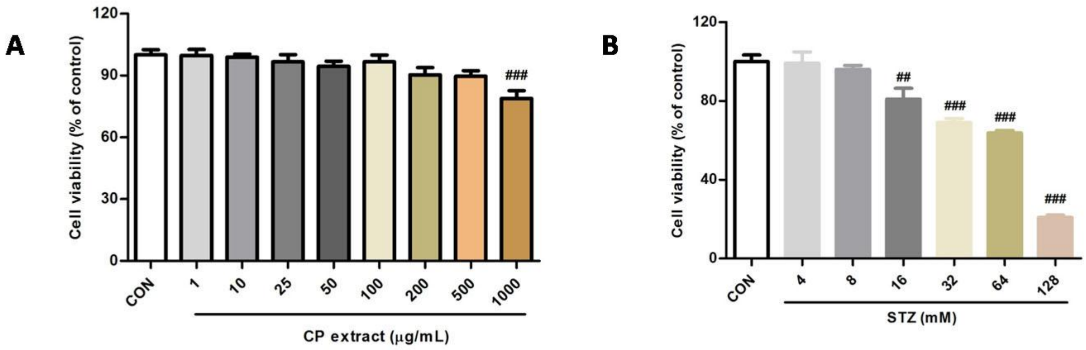

Fig S3 The cytotoxicity of CP extract and STZ on NIT-1 c.ells. **(A)** NIT-1 cells were incubated with differentcon centrations of CP extract (1-1000 μg/mL) for 24 h. **(B)** NIT-1 cells were incubated with different concentrations of STZ (4-128 mM) for 24 h. Cell viability was assessed by MTT assay. Data were expressed as mean ± SEM from three independent experiments. $^{\#\#}p < 0.01$ and $^{\#\#\#}p < 0.001$, compared with untreated control group.

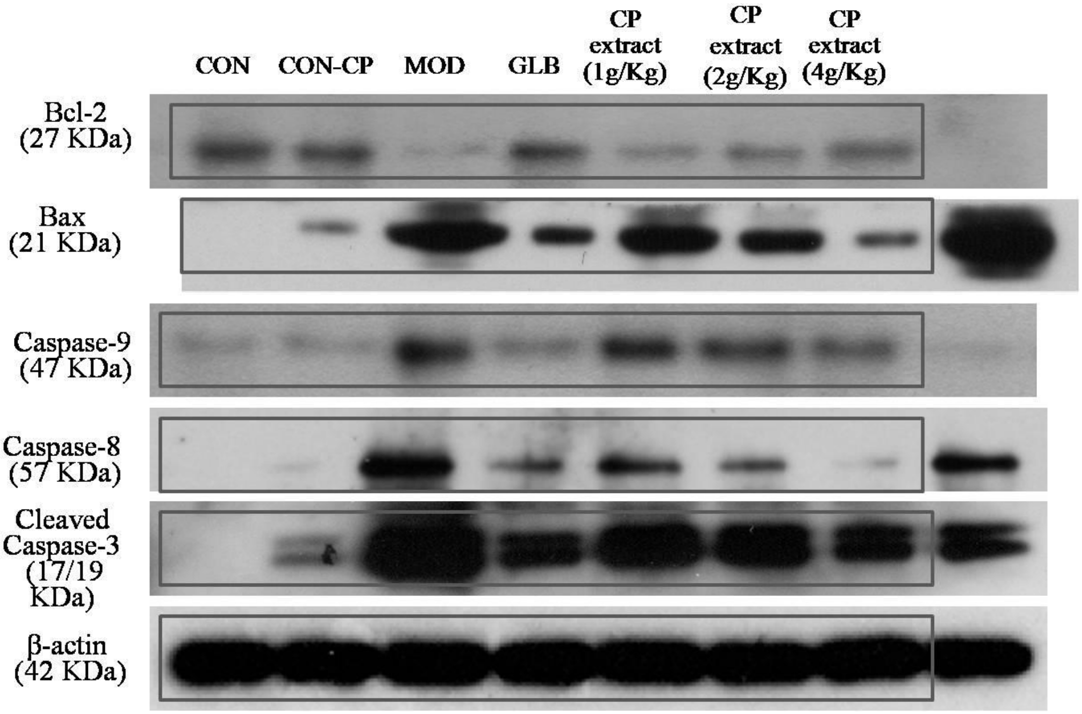

Fig S4 The full-length blots of Figure 4 (A).

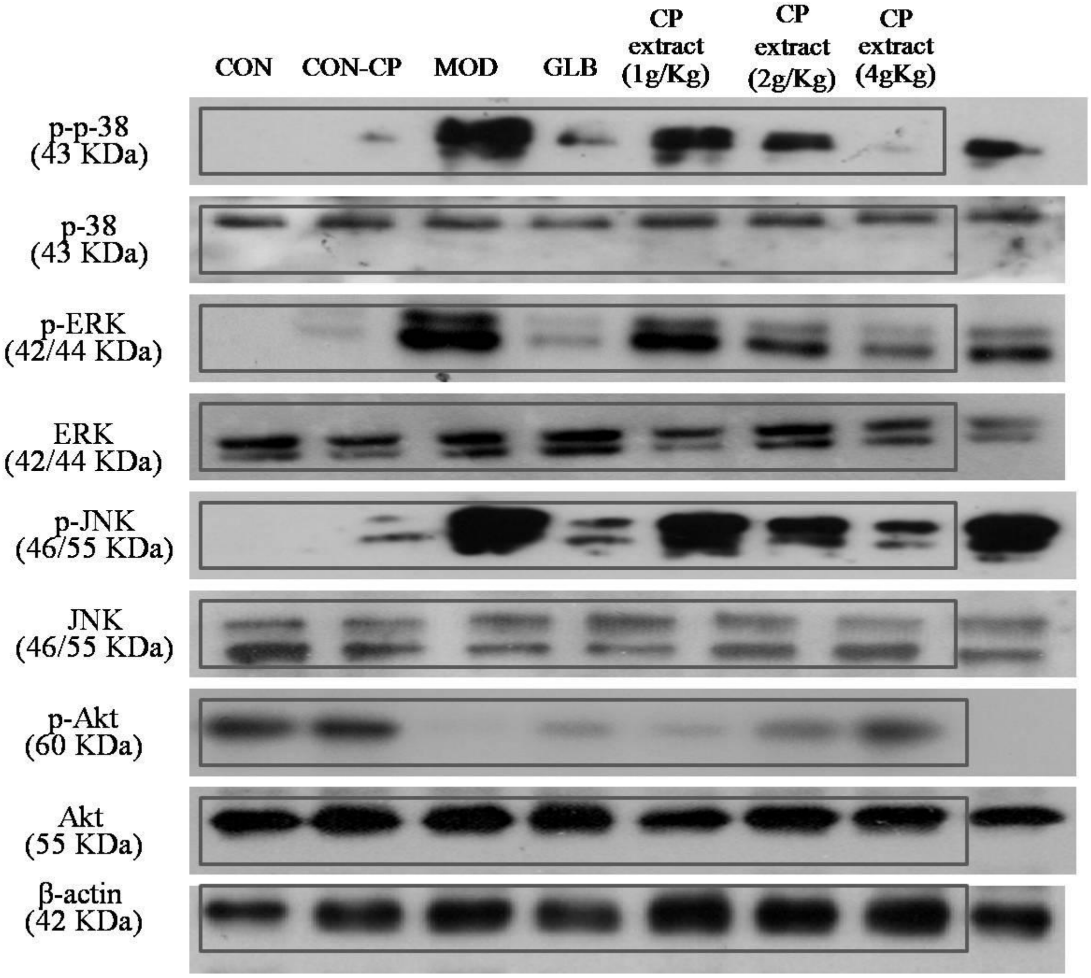

Fig S5 The full-length blots of Figure 5 (A).

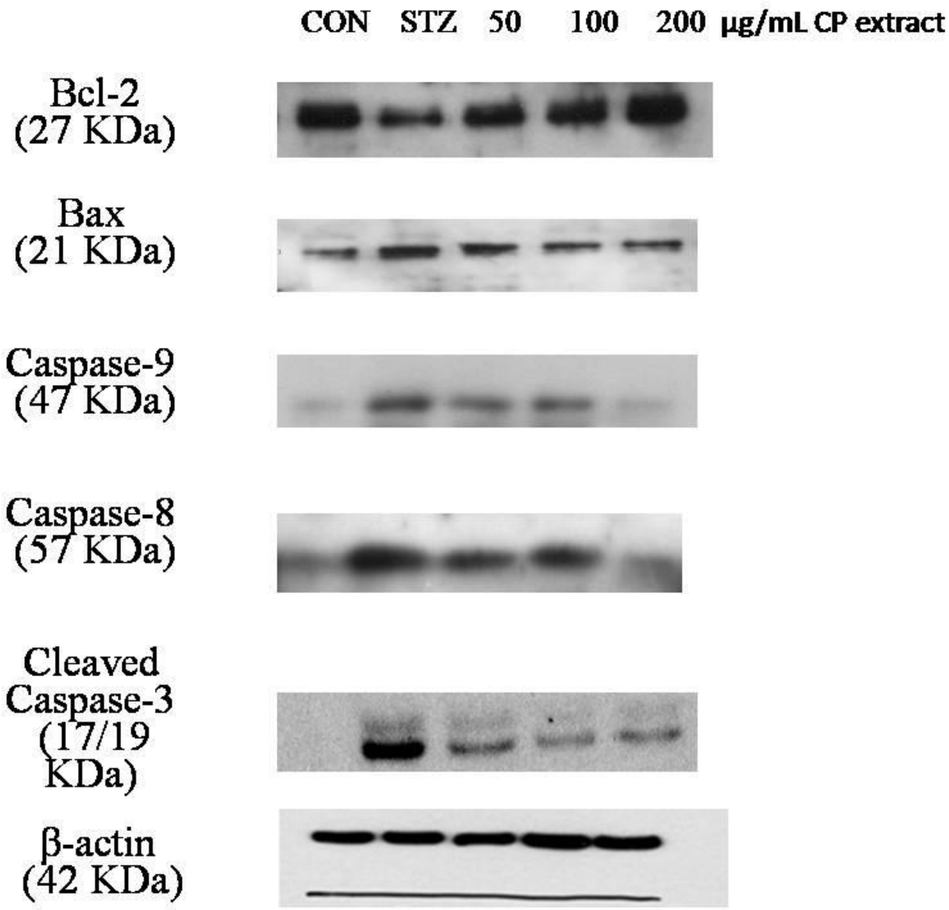

Fig S6 The full-length blots of Figure 6 (D).

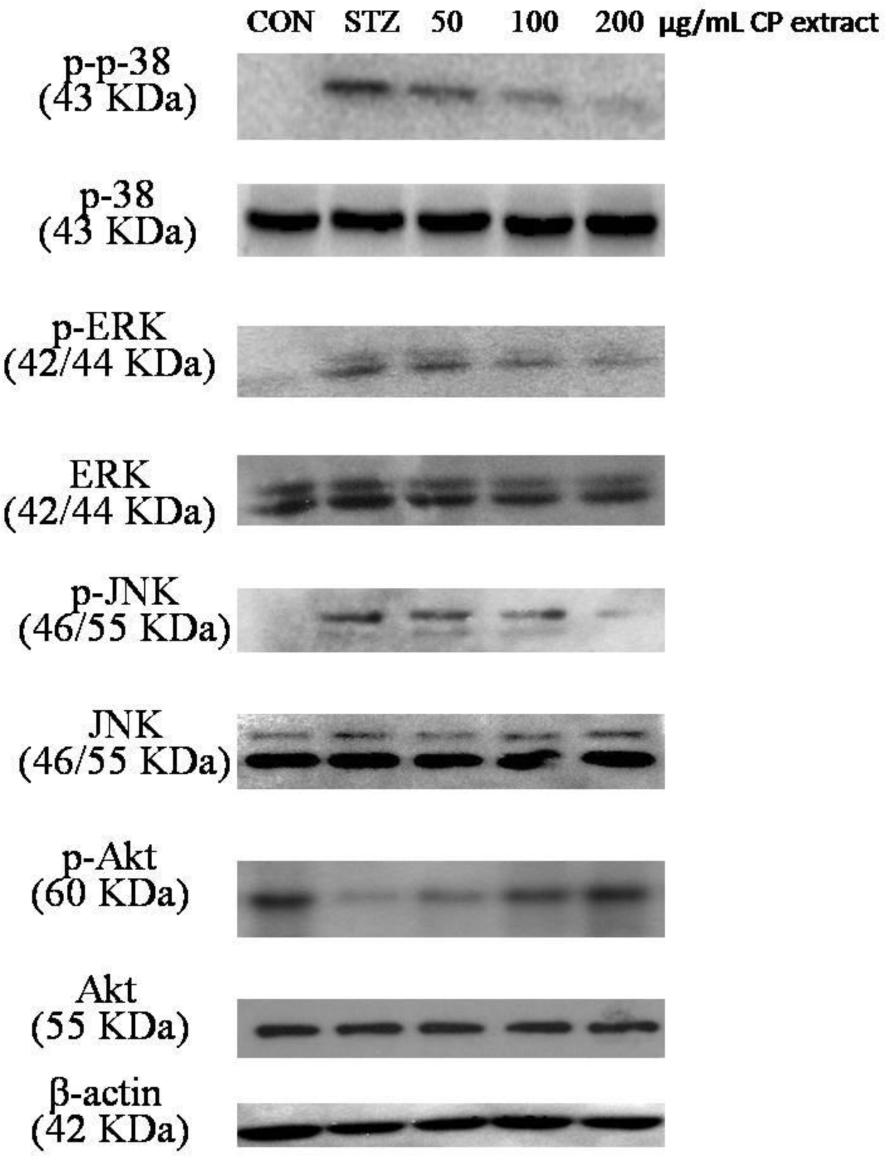

Fig S7 The full-length blots of Figure 7 (A).
